# *Porphyra tenera* Protects against PM_2.5_-Induced Cognitive Dysfunction with the Regulation of Gut Function

**DOI:** 10.3390/md20070439

**Published:** 2022-06-30

**Authors:** Seon Kyeong Park, Jin Yong Kang, Jong Min Kim, Min Ji Kim, Hyo Lim Lee, Jong Hyun Moon, Hye Rin Jeong, Hyun-Jin Kim, Min-Yu Chung, Ho Jin Heo

**Affiliations:** 1 Division of Applied Life Science (BK21), Institute of Agriculture and Life Science, Gyeongsang National University, Jinju 52828, Korea; pseonkyeong@kfri.re.kr (S.K.P.); kangjy2132@wikim.re.kr (J.Y.K.); myrock201@gnu.ac.kr (J.M.K.); minjee9790@gnu.ac.kr (M.J.K.); gyfla059@gnu.ac.kr (H.L.L.); 2020210043@gnu.ac.kr (J.H.M.); gpfls1428@gnu.ac.kr (H.R.J.); hyunjkim@gnu.ac.kr (H.-J.K.); 2 Korea Food Research Institute, Jeonju 55365, Korea; mic07002@kfri.re.kr; 3 Fermentation Regulation Research Group, World Institute of Kimchi, Gwangju 61755, Korea

**Keywords:** *Porphyra tenera*, PM_2.5_, cognition, inflammation, gut microbiota, sulfated galactan, mycosporine-like amino acids, chlorophyll derivatives

## Abstract

To evaluate the biological effects of *Porphyra tenera* (*P. tenera*), we tried to confirm the possibility that the intake of *P. tenera* could modulate cognitive and intestinal functions in PM_2.5_-induced cognitive decline mice. *P. tenera* attenuated PM_2.5_-induced learning and memory impairment through antioxidant and anti-inflammatory effects by regulating the mitochondrial function and TLR-initiated NF-κB signaling. In addition, *P. tenera* effectively alleviated Aβ production/tau phosphorylation by inhibiting the JNK phosphorylation. Also, the bioactive constituents of *P. tenera* determined the sulfated galactan, mycosporine-like amino acids (MAAs), and chlorophyll derivatives. Moreover, the bioactive compounds of *P. tenera* by gut fermentation protected against gut dysbiosis and intestinal tight junction damage with a decrease in inflammatory response and short-chain fatty acid production. Based on these results, our findings suggest that *P. tenera* with sulfated galactan and MAAs is a potential material for cognitive function improvement.

## 1. Introduction

Particulate matter ≤ 2.5 with an aerodynamic diameter (PM_2.5_) is a critical health hazard factor of air pollution. Inflammatory response and oxidative stress by PM_2.5_ are considered a critical mechanism of action for pulmonary and other health damages [1]. Many studies have demonstrated a correlation with respiratory and cardiovascular disorders [2,3]. Also, the correlation between PM_2.5_ and neurodegenerative or mental diseases such as Alzheimer’s disease (AD) has been recently reported. PM_2.5_ generates reactive oxygen species (ROS) in relevant organs and cells, and accelerates cognitive dysfunction by inducing oxidative damage in the central nervous system [2]. In addition, PM_2.5_ has a strong link between brain function and peripheral inflammation response. Systemic inflammation by PM_2.5_ could be initiated by the gastrointestinal tract as well as the pulmonary tract, such as the nasal cavity, bronchioles and alveolar spaces [4]. Systemic inflammation factors produced by PMs such as pro-inflammatory cytokines (interferon (IFN)-γ, interleukin (IL)-6 IL-1β, and tumor necrosis factor (TNF)-α), enter the brain through the blood-brain barrier (BBB) and activate the microglia. Furthermore, PM_2.5_ can directly reach the brain through the olfactory bulb, and it induces the destruction of the BBB by suppressing the expression levels of tight junction-related proteins [5,6]. The disrupted BBB can allow peripheral inflammatory or toxicity-associated substances to easily reach the brain [7]. Neuroinflammation by the multiple pathways of PM_2.5_ leads to damage of the synaptic, spatial, learning and memory functions [5]. According to a recent study, PMs such as heavy metals have easier access to gastrointestinal fluids than to the fluids in lung phase, and cause a serious imbalance of gut microbiota and biochemicals [8]. Therefore, it was suggested that the regulation of systemic inflammation could be the key for various PM-related diseases through gut function. It is necessary to study protective materials for PM_2.5_-induced cognitive decline, and our study has focused on protective materials for cognitive function by PM_2.5_-induced systemic inflammation.

Fat- and calorie-free red seaweed is being sought for commercial purposes, and its physiological activity makes it a very interesting source of functional ingredients [9]. *Porphyra tenera* (*P. tenera*; common name: laver) is a popular sea vegetable, and is the most common edible red seaweed parts of Asia such as China, Japan, and South Korea [10]. *P. tenera* is reported to have excellent nutritional value and includes vitamin B_12_, essential mineral elements and proteins, and has high blood pressure, high blood sugar, anti-inflammatory, immunomodulatory, neuroprotective, and skin anti-photoaging effects [9,11,12,13]. Despite the expected health benefits of *P. tenera*, not many in vitro/in vivo studies have been reported. The major compounds of *P. tenera* could show health effects through digestion and fermentation in the intestinal tract. In order to understand the health benefits of *P. tenera*, it is necessary to understand the process in the intestinal tract. For this reason, our study focused not only on the cognitive function but also the gut function (gut physiological character, microbiota modulation and short-chain fatty acids (SCFAs) level) by the administration of *P. tenera*.

## 2. Results

### 2.1. Ameliorating Effect on Learning and Memory Impairment

**Figure 1:**
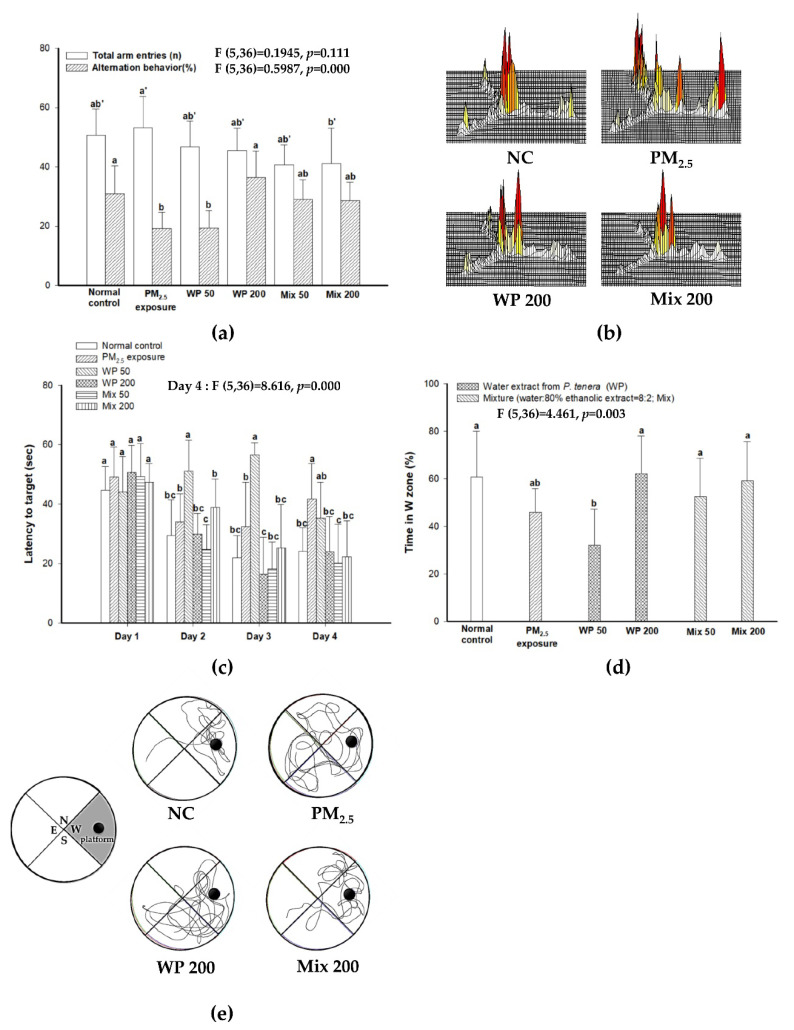
The ameliorating effect of *P. tenera* on PM_2.5_-induced cognitive decline. Total arm entries and alternation behavior (**a**) and the mice movements of each group (**b**) in the Y-maze test. Escape latency in the hidden-platform test (**c**), time spent in the W zone (**d**) and mice movement in the probe test (**e**) in the Morris water maze test. The data is shown as mean ± SD (*n* = 7), and different lowercase letters indicate a statistical difference (*p* < 0.05).

In the Y-maze test, the number of total arm entries of the PM_2.5_ group showed decreased mobility compared with the NC group, and the intake of the water extract from *P. tenera* (200 mg/kg of body weight, WP200 group) and a mixture (water extract: 80% ethanolic extract from *P. tenera* = 8:2, 200 mg/kg of body weight, Mix200 group) showed enhanced mobility. The PM_2.5_ (19.16%) group showed a decline in working spatial memory in alternation behavior compared with the normal control (NC; 30.93%) (Figure 1A). On the other hand, the administration of WP200 (36.44%) and Mix200 (28.50%) effectively ameliorated working spatial memory impairment. Indeed, as seen in Figure 1B, the mice in the PM_2.5_ group showed different movements in each arm. Meanwhile, the mice in the NC, WP200 and Mix200 groups indicated similar path tracing in each arm (Figure 1B).

The learning and memory function was confirmed using the Morris water maze test. During the four-day training period, the time to find the hidden platform decreased for all mice (Figure 1C). On the last day of training, the PM_2.5_ group (41.67 s) showed a higher latency time to find the platform than the NC group (24.09 s), while the WP200 (24.01 s) and Mix200 (22.24 s) groups showed significantly decreased latency time. In the probe test (Figure 1D,E), the PM_2.5_ group (46.02%) had decreased memory of the place (W zone) where the platform was located in contrast to the NC group (60.95%). The administration of the WP200 (62.21%) and Mix200 (59.38%) groups showed similar memory compared with the NC group.

### 2.2. Inhibitory Effect of Neuroinflammation

**Figure 2:**
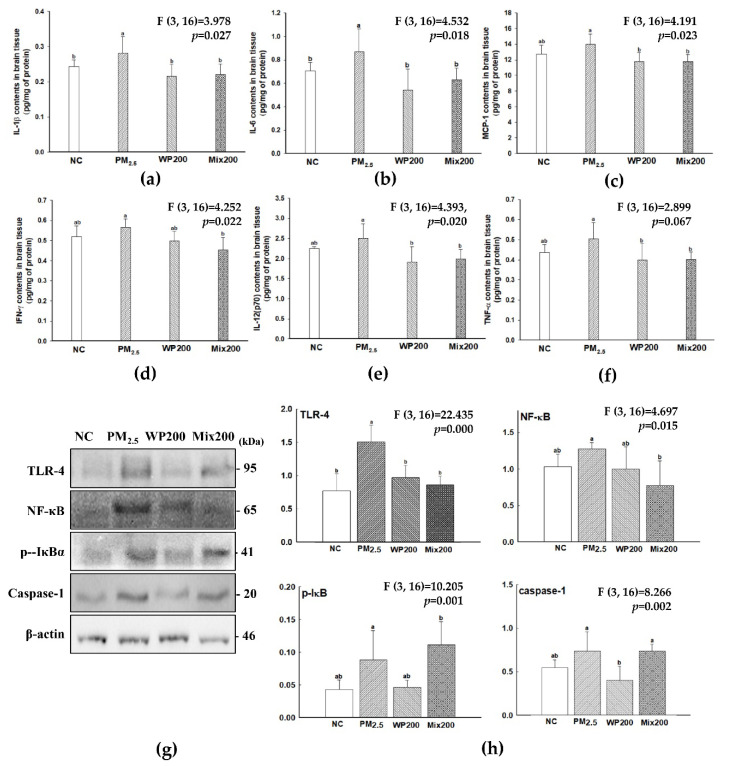
Inhibitory effect of *P. tenera* on PM_2.5_-induced neuroinflammation. Levels of pro-inflammatory cytokines such as IL-1β (**a**), IL-6 (**b**), MCP-1 (**c**), IFN-γ (**d**), IL-12(p70) (**e**), and TNF-α (**f**). Band images of western blot analysis (**g**) and the expression level of NF-Κb/inflammasome-related molecules (**h**). The data is shown as mean ± SD (*n* = 5), and different lowercase letters indicate a statistical difference (*p* < 0.05).

Pro-inflammatory cytokines (IL-6, TNF-α, MCP-1, IFN-γ, IL-12(p70)) increased in the brain by PM_2.5_ exposure, and WP200 and Mix200 effectively prevented the production of pro-inflammatory cytokines (Figure 2). The results of inflammation-mediated molecule (toll like receptor (TLR)-4, nuclear factor kappa-light-chain-enhancer of activated B cells (NF-κB), p-inhibitors of NF-κB (IκB) and caspase-1) analysis indicated that both WP200 and Mix200 were effective on TLR-4-initiated NF-κB/inflammation signaling by PM_2.5._

### 2.3. Antioxidant Effect of P. tenera

**Figure 3:**
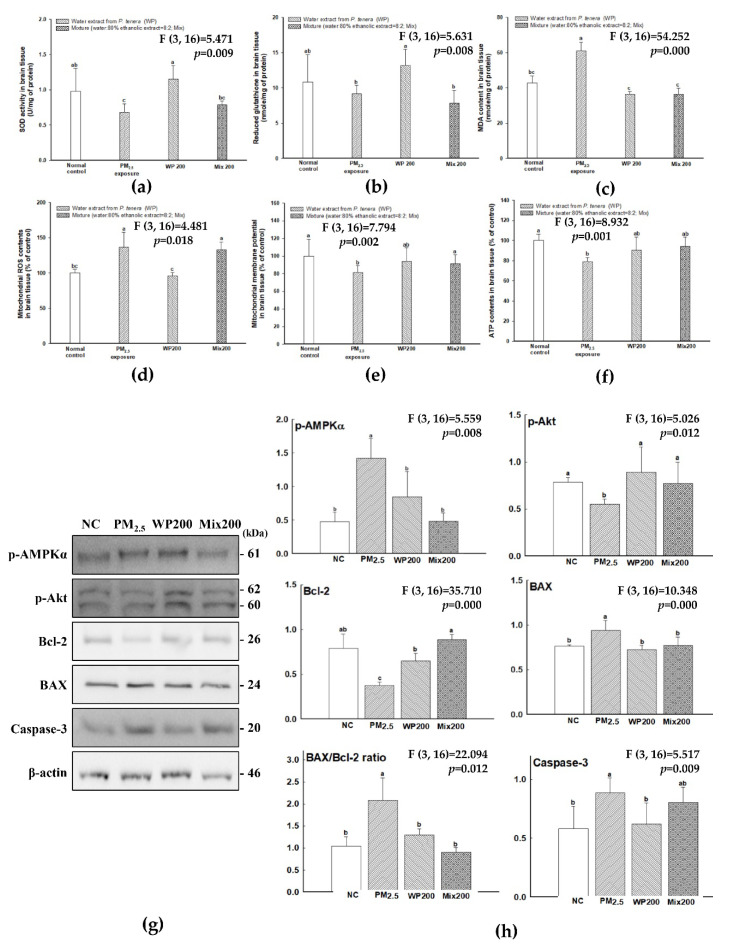
Antioxidant effect of *P. tenera* on PM_2.5_-induced oxidative stress. SOD (**a**), reduced GSH (**b**), and MDA (**c**) contents in brain tissue. Mitochondrial ROS content (**d**), MMP (**e**), ATP levels (**f**), band images of western blot analysis (**g**), and the expression level of apoptosis-related signaling molecules (**h**) in the brain. The data is shown as mean ± SD (*n* = 5), and different lowercase letters indicate a statistical difference (*p* < 0.05).

The antioxidant effect of WP and Mix was measured on PM_2.5_-induced oxidative stress in the brain, and the results are shown in Figure 3. Antioxidant molecules (such as superoxide dismutase (SOD) and reduced glutathione (GSH) contents decreased by PM_2.5_ compared with the NC group (Figure 3A,B), and the WP200 group showed relatively higher SOD and reduced GSH contents than the Mix200 group. On the other hand, malondialdehyde (MDA) content, an important marker of lipid peroxidation, showed that PM_2.5_ (61.08 μmole/mg of protein) induced lipid peroxidation compared to the NC group (42.94 μmole/mg of protein), while both the WP200 (36.44 μmole/mg of protein) and Mix 200 (36.63 μmole/mg of protein) groups effectively inhibited lipid peroxidation (Figure 3C).

PM_2.5_ exposure caused mitochondrial dysfunction by increasing ROS contents and decreasing mitochondrial membrane potential (MMP) and adenosine triphosphate (ATP) contents (Figure 3D–F). The intake of WP200 effectively enhanced the mitochondrial function, while Mix200 had an effect on the improvement of MMP and ATP content, but had no effect on the inhibition of ROS production. The molecules of mitochondria-mediated apoptosis signaling (p-AMP-activated kinase (AMPK)α, p-protein kinase B (Akt), B-cell lymphoma 2 (Bcl-2), and caspase-3) were analyzed, and PM_2.5_ led to an apoptosis-related mechanism in the brain (Figure 3G,H). The decrease in ATP content by PM_2.5_ expressed p-AMPKα. Furthermore, long-term p-AMPKα expression led to a decrease in p-Akt and Bcl-2 expression and an increase in BCL2 associated X (BAX) expression with mitochondrial dysfunction. Finally, the increase in the BAX/Bcl-2 ratio induced caspase-3 expression. The intake of WP200 and Mix200 effectively regulated the mitochondria-related apoptosis molecules.

### 2.4. Expression of Cognition-Mediated Protein

**Figure 4:**
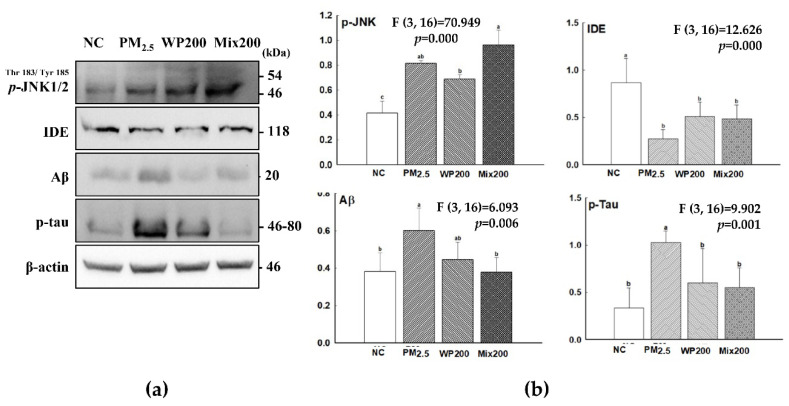
The regulation of *P. tenera* on PM_2.5_-induced cognitive dysfunction. Band images of western blot analysis (**a**), and the expression level of Aβ production/tau phosphorylation-related molecules (**b**) in the brain. The data is shown as mean ± SD (*n*=5), and different lowercase letters indicate a statistical difference (*p*<0.05).

Inflammatory response and oxidative damage in the brain lead to the expression of cognitive-related molecules (amyloid beta (Aβ) and tau phosphorylation). PM_2.5_-induced oxidative stress and inflammation caused p-c-Jun N-terminal kinases (JNK) expression, and then irritated Aβ production and tau phosphorylation (Figure 4). WP200 and Mix200 effectively regulated the cognitive-related molecules.

### 2.5. Main Compound Analysis

**Figure 5:**
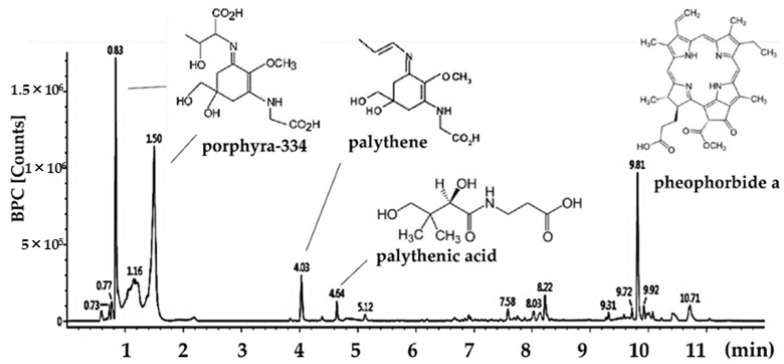
UPLC-MS chromatogram and structure of identified compound.

The total polysaccharide contents of *P. tenera* was detected as 46.23%, and its average molecular weight was confirmed to be 220.49 kDa (Table 1). The sulfate content was 43.25%, and monosaccharides were composed as follows: fucose (6.52%), rhamnose (7.83%), galactose (44.24%), xylose (23.18%), and other monosaccharides (18.23%). As a result, it was confirmed that the major polysaccharides of *P. tenera* are sulfated polysaccharide based on galactose as the main sugar backbone. Other major bioactive compounds in *P. tenera* were analyzed with the ultra-high performance liquid chromatography with quadrupole time-of-flight mass spectrometry (UPLC-QTOF MS)^2^ system (Figure 5 and Table 2). Identified compounds were confirmed by comparison with MS fragments from previous literature. Mycosporine-like amino acids (MAAs) including porphyra-334 isomers (0.85 and 1.58 min, *m*/*z* 347.1450; 303, 244, 227, 200, 186), palythene (4.36 min, *m*/*z* 285.1438; 241, 197), and palythenic acid (4.93 min, *m*/*z* 329.1333; 279, 253, 233, 205, 187, 150) were detected as major bioactive compounds, and the chlorophyll derivative pheophorbide a (9.92 min, *m*/*z* 593.2760; 533, 460, 447) was also identified [14].

### 2.6. Variation in Microbiome

**Figure 6:**
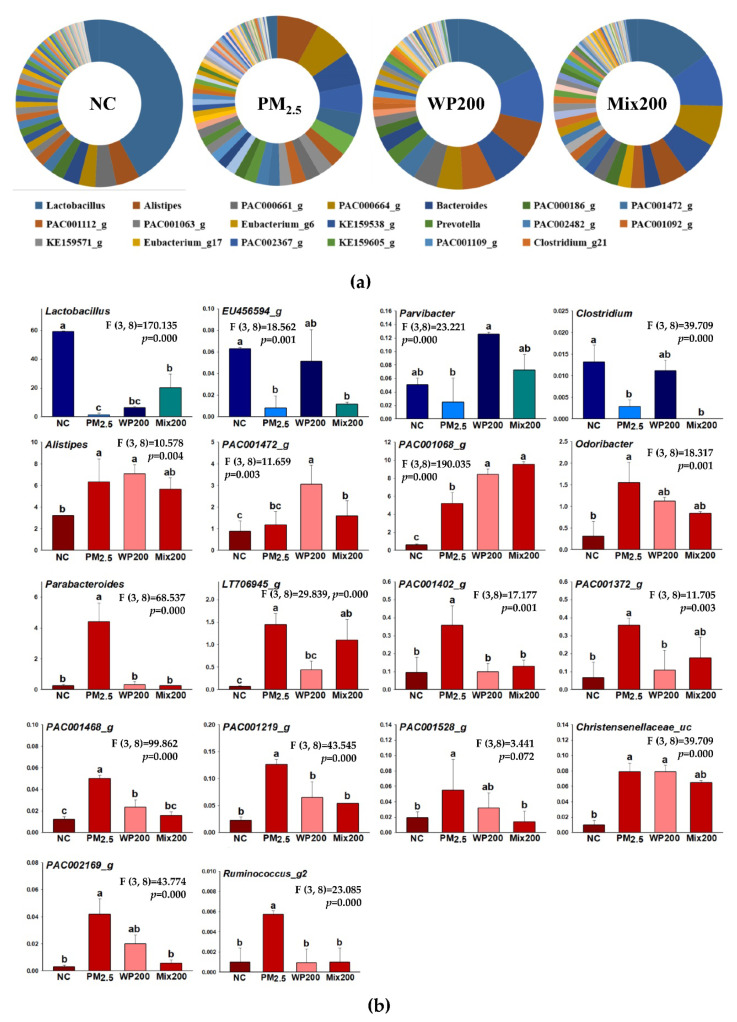
Variation in the gut microbiota by *P. tenera* in PM_2.5_-induced gut dysbiosis. The relative abundances of the genus in each group (**a**), and significant changes in some genera (**b**) in feces. The data is shown as mean ± SD (*n* = 3), and different lowercase letters indicate a statistical difference (*p* < 0.05).

Variation in the microbiome was analyzed, and the main changes were observed in genus-level community composition (Figure 6). The strains that showed a statistical difference in genus are shown in Figure 6B. The amounts of *Lactobacillus, EU456594_g*, *Paravibacter*, *Clostridium* decreased, and *Alistipes, PAC001472_g*, *PAC001068_g*, *Odoribacter*, *Parabacteroides*, *LT706945_g*, *PAC001402_g*, *PAC001372_g*, *PAC001468_g*, *PAC001219_g*, *PAC001528_g*, *Christensenellaceae_uc*, *PAC002169_g*, *Ruminococcus_g2* increased by PM_2.5_ exposure compared with the NC group. The intake of WP200 (*EU456594, Paravibacter, Clostridium*) and Mix200 (Lactobacillus, *Paravibacter*) effectively restored the healthy, beneficial gut bacteria. WP200 (*Odoribacter*, *Parabacteroides*, *LT706945_g*, *PAC001402_g*, *PAC001372_g*, *PAC001468_g*, *PAC001219_g*, *PAC001528_g*, *PAC002169_g*, *Ruminococcus_g2*) and Mix200 (*Alistipes*, *PAC001472_g*, *PAC001068_g*, *Odoribacter*, *Parabacteroides*, *LT706945_g*, *PAC001402_g*, *PAC001372_g*, *PAC001468_g*, *PAC001219_g*, *PAC001528_g*, *Christensenellaceae_uc*, *PAC002169_g*, *Ruminococcus_g*) significantly decreased in genus-level strains by PM_2.5_.

### 2.7. Expression of Tight Junction Proteins

**Figure 7:**
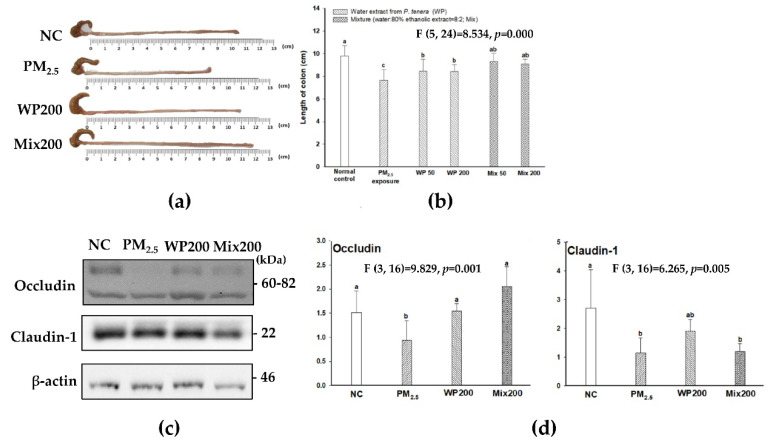
The protective effect of *P. tenera* against PM_2.5_-induced intestinal dysfunction. A representative image of the colon (**a**) and length of colon (**b**). Band images of western blot analysis (**c**) and the expression level of tight junctions-related proteins (**d**) in large intestine. The data is shown as mean ± SD (*n* = 5), and different lowercase letters indicate a statistical difference (*p* < 0.05).

The length of the colon was shortened by PM_2.5_, and the administration of WP200 and Mix200 effectively protected against these changes (Figure 7A,B). In addition, PM_2.5_ decreased the expression of intestinal tight junction (TJ) structural proteins (occludin and claudin-1). WP200 and Mix200 also protected TJ by regulating the expression occludin and claudin-1 (Figure 7C,D).

### 2.8. Inhibitory Effect of Inflammation in Gut and Blood Serum 

**Figure 8:**
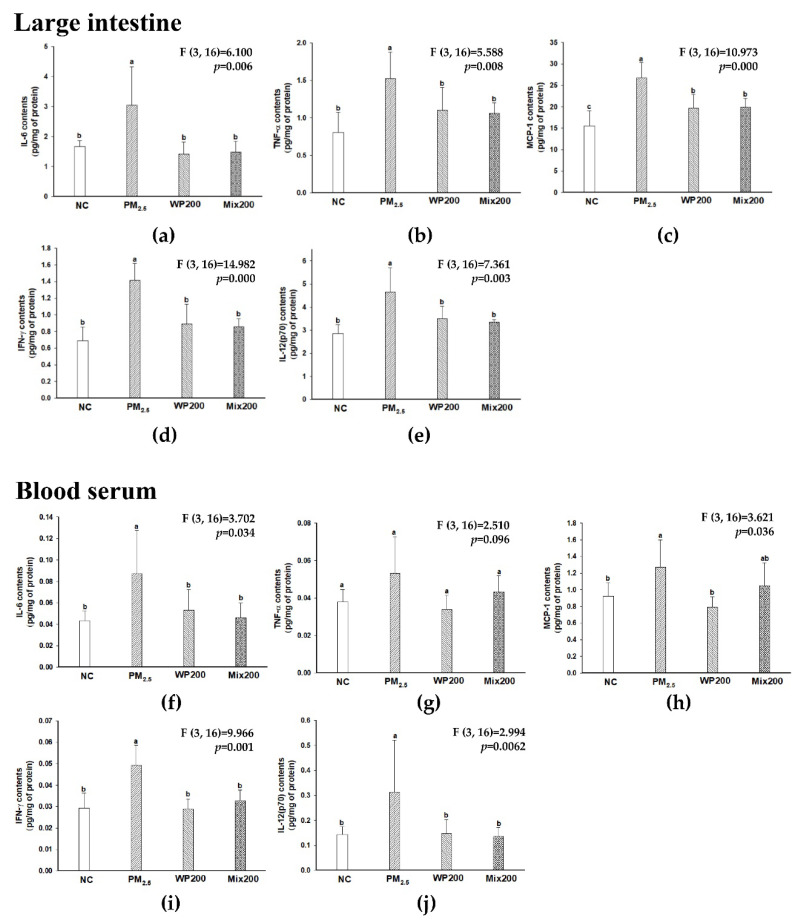
Inhibitory effect of *P. tenera* on PM_2.5_-induced inflammation in gut and blood serum. Levels of pro-inflammatory cytokines such as IL-6 (**a**), TNF-α (**b**), MCP-1 (**c**), IFN-γ (**d**), and IL-12(p70) (**e**) in large intestine. Levels of pro-inflammatory cytokines such as IL-6 (**f**), TNF-α (**g**), MCP-1 (**h**), IFN-γ (**i**), and IL-12(p70) (**j**) in blood serum. The data is shown as mean ± SD (*n* = 5), and different lowercase letters indicate a statistical difference (*p* < 0.05).

The results of pro-inflammatory cytokine contents (IL-6, TNF-α, MCP-1, IFN-γ, IL-12(p70), and IL-1β) in gut and blood serum are shown in Figure 8. The PM_2.5_ group increased pro-inflammatory cytokines in gut and blood serum more than the NC group, while the WP200 and Mix200 groups effectively decreased cytokines. PM_2.5_ exposure was confirmed to induce inflammatory responses in the gut (digestive tract) and induced systemic inflammation by moving the pro-inflammatory cytokines into the blood.

### 2.9. Fecal Short-Chain Fatty Acids (SCFAs) Analysis

**Figure 9:**
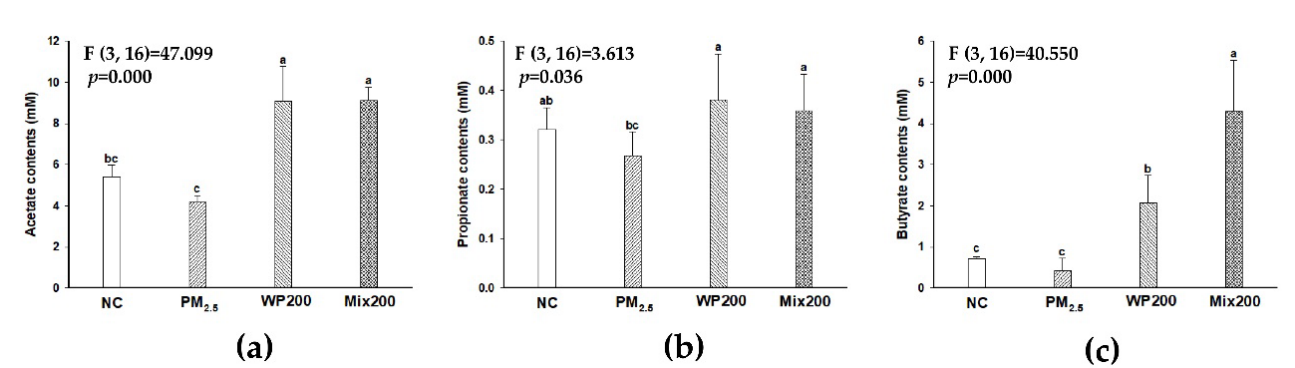
Analysis of fecal short-chain fatty acids (SCFAs) level including acetate (**a**), propionate (**b**), and butyrate (**c**). The data is shown as mean ± SD (*n* = 5), and different lowercase letters indicate a statistical difference (*p* < 0.05).

PM_2.5_-exposed mice, acetate (4.16 mM) and propionate (0.27 mM) decreased, and butyrate (0.42 mM) showed no statistical difference compared with the NC group (acetate; 5.39 mM, propionate; 0.32 mM, and butyrate; 0.70 mM) (Figure 9). WP200 (acetate; 9.08 mM, propionate; 0.38 mM, and butyrate; 2.07 mM) and Mix200 (acetate; 9.11 mM, propionate; 0.35 mM, and butyrate; 4.30 mM) significantly increased SCFAs contents, and both WP200 and Mix200 exhibited a higher content of SCFAs than the NC group.

## 3. Discussion

Air pollution can cause cognitive dysfunction as a result of inflammatory response, oxidative stress of the nervous system, protein modification, and cerebral vascular-barrier disorders in the brain, and then increase the risk of Alzheimer's disease and vascular dementia [15]. In our results, long-term PM_2.5_ exposure for 12 weeks caused learning and memory impairment in mice, and the intake of WP and Mix effectively protected against cognitive dysfunction (Figure 1). So far, there have been no reports on the cognitive function-related effects of *P. tenera*. However, the potential for cognitive improvement of red algae has been expected due to their functional ingredients such as polysaccharides, phenolics, lipids, proteins and carotenoids [16]. Edible red algae *Gracilariopsis chorda* protected cultured hippocampal neurons by enhancing early neuronal differentiation and axon/dendritic arborization [17]. Red algae marine *Meristiella echinocarpa* ameliorated the anxiety-like behavior and locomotion in an open field test, and modulated the striatum, hippocampus, and prefrontal cortex regions of the brain through GABAergic and glycinergic pathways with antioxidant and anti-convulsant effects [18]. These reports suggest that *P. tenera* as an edible red algae has the potential to enhance cognitive function.

In our previous study, we confirmed that PM_2.5_-induced inflammation was initiated in TLR-4 in the brain and lungs [19]. TLRs are an innate immune receptor in the brain and recognize multiple endogenous ligands that are released as cellular damage signals [20]. Among the various TLR subtypes, TLR-4 activates the NF-κB signaling pathway [21]. TLR-4 expression is related to phosphorylation of the IκB, and break the NF-κB/IκB complex [22]. As a result, NF-κB was transferred to the nucleus, and the inflammation/apoptosis-related gene triggered pro-inflammatory cytokine synthesis such as IL-6, IL-1β and TNF-α. In addition, NF-κB triggered NLRP-3 expression and induced inflammasome production. Inflammasomes activate caspase-1 and induce IL-1β release. As shown in Figure 2, *P. tenera* alleviated PM_2.5_-induced inflammation response by suppressing the TLR-4-initiated NF-KB/inflammasome signaling pathway. As a result, pro-inflammatory cytokines were effectively inhibited in the brain. Astaxanthin, a red-colored phytonutrient carotenoid found in marine organisms, was reported to have a potential beneficial effect on PM_2.5_ exposure in the brain through the protective effect of cultured glial and BV-2 microglial cells [23]. Astaxanthin effectively regulated PM_2.5_-induced inflammatory response and neurotoxicity *via* the inhibition of pro-inflammatory markers (IL-1β, IL-6, TNF-α, iNOS, triggering receptor expressed on myeloid cells 2, heme oxygenase-1) in BV-2 microglial cells, and the regulation of proinflammatory M1 and disease-associated microglia phenotype in cultured glial cells. In addition, Senevirathne et al. (2010) reported that enzymatic extracts of *P. tenera* effectively inhibited H_2_O_2_-induced DNA damage and LPS-induced NO synthesis in RAW264.7 cells [24]. Furthermore, the water extract of *P. tenera* significantly protected nasal epithelial (RPMI-2650), lung epithelial (A549), and brain neuroblastoma (MC-IXC) cells against PM_2.5_, and effectively alleviated the inflammatory response of the lungs through the regulation of pro-inflammatory cytokines (IL-1β, TNF-α and IL-6) in short-term PM_2.5_-exposed mice for four weeks [25]. Some studies have reported that the polysaccharides, proteins and peptides from *Porphyra* species have an immunomodulatory effect. Porphyran, a representative of the *Porphyra* species, reportedly has cognitive function-related neuroprotective effects. According to Liu et al. (2019), oligo-porphyrin effectively protected PC12 neuronal cells as an inflammatory mediator by regulating pro-inflammatory cytokines (IL-1β, IL-6, and TNF-α) [26]. Also, porphyran from *Porphyra yezoensis* showed a neuroprotective effect in cerebral ischemia-reperfusion injured rats by regulating NF-κB signaling pro-inflammatory cytokines with an antioxidant effect (SOD, CAT, and reduced GSH) [27]. Based on our results and the previous literature, *P. tenera* could be a novel material for PM_2.5_-induced diseases by regulating the inflammation response.

It is already well known that oxidative stress is closely related to air pollution and health damage, and the results of a meta-analysis indicated the differences in oxidized DNA and lipids in blood, urine, and airway between humans exposed to air pollution and unexposed humans [1]. Environmental toxins such as O_3,_ PM_2.5_, CO, and NOx play a role in the development of neurodegenerative disease by inducing oxidative damage in the brain [2]. Therefore, we tried to confirm the antioxidant effect of *P. tenera* in the brain. Changes in SOD, reduced GSH, catalase activity and lipid peroxidation appear as indicators of antioxidant defense system balance [28]. In the antioxidant system, mitochondria are a major organelle regulating oxidative stress in the brain. Mitochondrial membrane depolarization and excessive ROS production cause mitochondria dysfunction, and this leads to cell death [29,30]. The signal of cell damage and death is associated with inactivate anti-apoptotic Bcl2 proteins and BAX as pro-apoptotic Bcl-2 family proteins, and the BAX complex in the mitochondria membrane was induced to cytochrome c into cytosol. The released cytochrome c is related to apoptosome formation and caspase-3 activation [26]. Therefore, natural phenolic compounds in plants with a strong antioxidant effect such as quercetin and N-acetyl-cysteine have been suggested as drugs for cognitive decline by activating the mitochondrial function [31]. Meanwhile, seaweed phenolic compounds have been reported to play a critical role in antioxidant activity and lipid peroxidation [32,33,34]. However, various red algae extracts including *P. tenera* show an antioxidant effect (e.g., hydroxyl radical, superoxide anion, hydrogen peroxide, and DPPH free radical), and there was no correlation with phenolic compounds [35]. Many researchers have suggested that proteins (phycoerythrin), polysaccharides (porphyran), and MMAs (asterina-330, shinorine, palythine, palythinol and porphyra-334) from *Porphyra spp.* are associated with antioxidant activity [36]. The polysaccharide from *Porphyra yezoensis* repaired oxalate-injured renal cells by activating the mitochondrial function with the inhibition of ROS production, intracellular Ca^2+^, and loss of MMP. In addition, oligo-porphyran alleviated behavioral deficits in PD mice by regulating the phosphoinositide 3-kinase (PI3K)/Akt/Bcl-2 signaling pathway [37]. In our results, *P. tenera* treatment suppressed the BAX/BCl-2 ratio and inhibited caspase-3 activation in the brain by alleviating PM_2.5_-induced mitochondrial dysfunction (Figure 3).

Excessive oxidative stress and inflammatory response modified brain proteins and caused mild cognitive dysfunction and AD. In addition, some research has reported that PM_2.5_ is associated with the development of AD and AD-related neuropathology [5,6]. Aβ and tau are considered specific biomarkers of AD [38]. JNK, a key cellular regulator, is related to brain functions in development, memory formation, and brain tissue repair [39]. JNK is phosphorylated by various stress factors such as cytokines, growth factors, oxidative stress, and unfolded protein response signals [40]. Excessive JNK phosphorylation leads to inflammation, synaptic dysfunction, and cognitive deficits [39]. Oxidative stress is associated with the Aβ production mechanism through insulin signaling. p-JNK leads to p-IRS, and then insulin-degrading enzyme (IDE), which degrades Aβ as well as insulin, is inactivated and Aβ production signaling is activated [41]. In addition, p-JNK was associated with tau phosphorylation by downregulating GSK-3β signaling. Therefore, the inhibition of p-JNK plays a role in cognitive function by down-regulating Aβ production and tau phosphorylation. The inhibitory effect of red algae including *P. tenera* on Aβ production and tau phosphorylation has yet to be studied. The possibility of using some red algae as anti-amyloidogenic agents has been reported. The hybrid carrageenan-like sulphated galactan from *Furcellaria lumbricalis* inhibited beta-site amyloid precursor protein cleaving enzymes with immunomodulatory activity, and terpenoid-rich *Gelidiella acerosa* extract attenuated Aβ_25-35_ production and aggregation [42]. However, the detailed mechanism of anti-amyloidogenic activity of red algae was not determined. In our study, the ameliorating effect of *P. tenera* on PM_2.5_-induced cognitive impairment led to lower tau phosphorylation and amyloid-beta production with the regulation of oxidative stress and inflammation (Figure 4). From this point of view, *P. tenera* has potential as a material for protection against cognitive impairment. However, our study is limited in that it analyzed the whole brain, not specific regions of the brain such as the hippocampus and prefrontal cortex.

The strong physiological activity of *Porphyra* species has been reported to be from polysaccharides, proteins, lipids, and minerals [43]. Among the various bioactive compounds, we identified sulfated galactan and MAAs as major compounds of *P. tenera* (Figure 5, Table 1 and Table 2). Seaweed consists of approximately 70% polysaccharides, and contains dietary fibers that are fermented only by intestinal microbes in the large intestine [44]. The absorbable final fermented products (i.e., short-chain fatty acids) play an essential role in the immune system. Recently, it was reported that the sulfated polysaccharide from *Porphyra haitanensis* modulated the proliferation of lymphocytes and regulated inflammatory markers such as TNF- and IL-10, CD4+ splenic T lymphocytes, dendritic cells and Tregs in mice [45]. In particular, the sulfated galactans mainly found in red algae and so-called porphyran have been found in *Porphyra* species. Porphyran and their oligomers are reported to have bioactive activity such as antioxidative, anti-cancer, anti-inflammatory, and hypolipidemic effects [43]. Moreover, *P. tenera* contains 10% porphyran with approximately 35% water-soluble polysaccharides. Porphyran from *Pyropia yezoensis* suppressed LPS-induced immune activity by competitive binding in spleen dendritic cells of mice [46]. Porphyran has potential pharmaceutical applications based on anti-allergic, anti-viral, and immunomodulating effects, metallic adsorption ability and improvement of microbiota. In addition, sulfated galactan is known to remove toxic heavy metals (Cd^2+^, Cu^2+^, Zn^2+^, Pb^2+^, Cr^3+^, and Hg^2+^) by biosorption [47], and its ability is expected to have physiological effects by binding to PM_2.5_ in our results.

MAAs are water-soluble and low molecular-weight molecules produced by the shikimate and pentose phosphate pathways [48]. Recently, MAAs have been suggested as novel antioxidants based on their inhibitory effects against the strongest UVR-induced oxidative stress [48]. Although MAAs have strong physiological activity in marine organisms, the various properties and mechanism of MAAs has not been reported. Previous studies have focused on photo-protective effects such as skin aging including anti-oxidative and anti-inflammatory effects against UV [49]. MAAs (Porphyra-334 and shinorine) extract from *P. tenera* effectively inhibited skin aging by enhancing procollagen production in UV irradiation exposed mice, and the protective effect of MMAs was related to anti-inflammation by reducing TNF-α with the regulation of the NF-κB and MAPK signaling pathway [13]. Importantly, it has been reported that MMAs, water-soluble molecules with 300–350 Da, have the possibility to easily cross the BBB and have a direct effect on the brain [50].

Pheophorbide a is one of the main chlorophyll derivatives that is directly absorbed in the intestine or produced in the liver, and can affect various organs by systemic circulation in mice and rabbit [51]. Pheophorbide a and pheophytin a from *Saccharina japonica* exhibited an anti-inflammatory effect by regulating NO, iNOS and COX-2 in LPS-induced RAW264.7 cells [52]. Moreover, pheophorbide an isolated from *Solanum diflorum* was suggested as one of the major compounds for the inhibition of the NF-κB mechanism in the ears of ICR mice [53]. Based on the previous reports and our results, *P. tenera* containing sulfated galactan, MAAs, and pheophorbide a as major bioactive compounds suggested potential novel applications for PM_2.5_-related disorders.

Interestingly, a recent study reported that PMs have easier access to gastrointestinal fluids rather than the respiratory tract, and that the intestinal microbiome and their metabolites are closely correlated with PMs. PM_2.5_ reaches the intestine through the gastrointestinal tract and causes microbiome changes [54]. The gut microbiota is a complex ecosystem that regulates immune system homeostasis and is related to various diseases (e.g., obesity, type 2 diabetes, non-alcoholic fatty liver, and cardiovascular and nervous system disorders) [44]. In particular, gut changes have been suggested as important factors influencing central nervous system diseases [5]. Gut microbiota dysbiosis in animals and humans has been implicated in behavioral and neurologic pathologies such as depression and AD. Therefore, the gut microbiota plays a critical role in host health, and materials for gut health are necessary. Carbohydrates and polysaccharides from our daily diet reach the gut through the gastrointestinal tract without being digested, and are fermented by commensal bacteria [55]. Moreover, sulfated galactan, a major compound containing 46.23% of *P. tenera* (Table 1)*,* has high molecular weight, so it is difficult to absorb directly in the intestine because it cannot be digested by human intestinal enzymes. However, it has potential as a material for prebiotics. Therefore, we evaluated the gut function (gut microbiome and their metabolites) with *P. tenera*. In our results (Figure 6), the administration of Mix increased the genus *Lactobacillus* as beneficial bacteria, and WP increased the genus *Clostridium* as butyrate-producing bacteria. On the other hand, the genera *Alistipes*, *Odoribacter*, *Paraterodes*, *and Ruminococcus_g2*, harmful bacteria from increased PM_2.5_ exposure, were effectively suppressed by *P. tenera*. More importantly, the correlation between gut bacteria and cognitive function has been reported in behavior and depression [56]. This correlation was associated with damage to gut permeability by intestinal bacteria or their metabolites, and systemic inflammation caused the release of pro-inflammatory cytokines and toxic compounds through damaged gut tight junctions [57]. Indeed, *Alistipes* and *Odoribacter* at the genus level are reported to increase in APP/PS1 mice and 5xFAD and AD patients [58]. These bacteria are known to be associated with the inflammatory response and damage to gut tight junctions. Although the correlation between changes in the microbiota by PM_2.5_ and cognitive dysfunction is still clear, changes in gut bacteria such as *Lactbacillus* and *Alistipes* at the genus level may have affected cognitive decline based on the previous literature [57]. *P. tenera* is a potential material that can protect not only gut health but also brain function by restoring the gut microbiota.

Recently, there has been increasing evidence that PM_2.5_ causes gut dysfunction through changes in the metabolic profiles of mice, and gut injury is closely connected to systemic inflammatory reactions [54]. The tight junction barrier is important for intestinal homeostasis as a frontline defense barrier and is involved in the regulation of immune responses by preventing antigens or pathogens [59]. Gut dysbiosis leads to tight junction damage and releases toxic chemicals such as pro-inflammatory cytokines into the blood, which causes systemic inflammation and various diseases. Systemic inflammation has been a focus to explain the correlation between PM_2.5_ and brain dysfunction [5]. In particular, PM_2.5_ causes the risk of neurodegenerative disease linked to brain inflammatory responses. Pro-inflammatory cytokines reaching the brain through the blood lead to the activation of microglia, and inflammatory signals in the brain are activated [60]. *Laminaria japonica,* including sulfated polysaccharide, restored LPS-induced loss of monolayer permeability by increasing the expression of occludin and then effectively attenuated NO and IL-6 production in Caco-2 cells [61]. Although the mechanism of PM_2.5_ and systemic inflammation or neuroinflammation is not fully understood, *P. tenera* effectively regulated pro-inflammatory cytokines in the gut, blood serum, and brain (Figure 8). These results suggest that the inhibition of systemic inflammation by preventing tight junction damage may have an influence on the protective effect against PM_2.5_-induced cognitive dysfunction. In addition, a significant increase in IFN-γ and IL-12, which are related to Th1 cells, was observed in the gut, blood serum, and brain after PM_2.5_ exposure in our results (Figure 2 and Figure 8). As another pathway for the PM_2.5_-related inflammation mechanism, the regulation of macrophage activation could be proposed. Macrophages are critical immune modulators, and are regulated by T cells [62]. Recently, it was reported that PM_2.5_ treatment increased cytokine mRNA expression such as IFN- γ, IL-4, IL-10, IL-17 and IL-21 in human CD4+ and CD8+ T cells, and the results demonstrated that PM_2.5_ exposure induced macrophage-dependent inflammation characterized by increased Th1/Th17-related cytokines secretion [63]. Together, our results suggest that PM_2.5_ and the inflammation response in the gut, blood serum, and brain might be related to the activation of macrophages by T cells. Although the mechanism of PM_2.5_ and systemic inflammation or neuroinflammation is not fully understood, *P. tenera* effectively regulated pro-inflammatory cytokines in the gut, blood serum, and brain (Figure 8).

Polysaccharides from marine algae are used as prebiotics for beneficial gut bacteria, and produce metabolites such as oligosaccharides and SCFAs [64]. In particular, SCFAs play an important role in the maintenance of the gut and immune homeostasis in the gut through the activation of G-protein-coupled receptors [65]. Also, SCFAs play a key role in microbiota–gut–brain communication by crossing the BBB, and effectively modulate neuroinflammation by regulating the maturation and function of microglia [66,67]. In particular, acetate suppressed pro-inflammatory cytokines (IL-1β, IL-6, and TNF-α) through the regulation of MAPK/NF-κB pathways, and butyrate alleviated neurological deficits, oedema, neuron damage and BBB impairment by occludin and ZO-1 expression in the brain [66,68]. According to a study by Kim et al. (2020), *P. tenera* extract alleviated harmful bacteria such as *Helicobacter*, *Mucipirillum*, and *Parasutterella*, and *Clostridium_XIVb*, and butyrate-producing bacteria by lactose fermentation were effectively increased in dextran sodium sulfate (DSS)-induced gut dysbiosis mice [44]. Also, the alteration of gut microbiota from *P. tenera* extract effectively alleviated colitis symptoms such as body weight loss, colon length loss, and diarrhea, and also attenuated the mRNA expression of pro-inflammatory cytokines (TNF-α, IL-6, IL-1β, and COX-2). In our results, the administration of *P. tenera* indicated gut health-promoting effects by preventing physical changes (length of colon) and intestinal inflammation with the regulation of gut microbiota, tight junction-related protein expression and SCFAs production as beneficial metabolites. Therefore, *P. tenera* could be a potential functional agent for cognitive function based on its gut health-promoting effects by sulfated galactan or direct effect by MAAs as materials that pass the BBB. Finally, because our study used only male mice, there are limitations regarding sex. Thus, the health benefit effects may differ according to sex.

## 4. Materials and Methods

### 4.1. Chemicals

PM_2.5_ was purchased from Powder Technology Inc. (Arizona Test Dust, Arden Hills, MN, USA). Thiobarbituric acid, trichloroacetic acid, phosphoric acid, digitonin, and all other chemicals used were purchased from Sigma-Aldrich Chemical Co. (St. Louis, MO, USA). Anti-p-IκB-α (sc-8404), anti-Bcl-2 (sc-509), anti-BAX (sc-7480), anti-caspase-1 (sc-392736), anti-β-amyloid (sc-28365), anti-p-Tau (sc-12952), anti-p-Akt1/2/3 (sc-101629), anti-p-JNK (sc-6254), anti-IDE (sc-393887), anti-TLR-4 (sc-52962), and anti-β-actin (sc-69879) were purchased from Santa Cruz Biotechnology (Santa Cruz, CA, USA), and anti-NF-κBp65 (#6956) and anti-p-AMPKα (#2531), and secondary antibodies were purchased from Cell Signaling Technology (Danvers, MA, USA). Anti-caspase-3 (CSB-PA05689A0Rb) was purchased from Cusabio Biotech (Wuhan, China).

### 4.2. Preparation of P. tenera Extract

*P. tenera* was obtained from Wando (Jeollanamdo, Korea) in February 2018. *P. tenera* was washed and lyophilized using a vacuum-tray freeze dryer (Operon, Gimpo, Korea). Lyophilized *P. tenera* was extracted with distilled water and 80% ethanol at 40 °C for 2 h. Each extract was filtered through Whatman No. 2 filter paper (Whatman International Limited, Kent, UK) under reduced pressure. Water extract from *P. tenera* (WP) was directly lyophilized without concentration. The 80% ethanolic extract was concentrated using a rotary vacuum evaporator (N-1000; Eyela Co., Tokyo, Japan) and lyophilized. Lyophilized extracts were stored at −20°C until use [25]. A mixture from *P. tenera* (Mix) was made by mixing with water extract and 80% ethanolic extract at a ratio of 8:2.

### 4.3. Animal Experimental Design

All animal experimental protocols were performed in accordance with the Policy of the Ethical Committee of Ministry of Health and Welfare (Korea), and approved by the Institutional Animal Care and Use Committee of Gyeongsang National University (certificate: GNU-180927-M0050). Male six-week old BALB/c mice were purchased from Samtako (Osan, Korea) and maintained in a pathogen-free environment with a 12 h light/dark cycle, 55% humidity, and 22 ± 2 °C temperature. Mice were freely fed a normal diet and drinking water during the entire experiment. The mice were randomly divided into six groups: a normal control (NC) group, a PM_2.5_-exposed group (PM_2.5_), WP groups (50 and 200 mg/kg of body weight (mpk); WP 50 and WP200), and Mix groups (50 and 200 mpk; Mix50 and Mix200). The water extract and 80% ethanolic extract were dissolved using drinking water, and the mixture was mixed with water extract and 80% ethanolic extract at a ratio of 8:2. The dissolved WP and mixture were administrated with a feeding needle once a day before PM_2.5_ exposure (500 μg/m^3^ concentration in whole-body exposure, 5 h/day, 5 days/week for 12 weeks) [19].

### 4.4. Whole Body Exposure of PM_2.5_

PM_2.5_ was dissolved using distilled water and sonicated for 30 min. The mice freely moved in a whole-body chamber (276 (w) × 420 (d) × 247 (h) mm). PM_2.5_ was sprayed into a mixing chamber through a mist generator at a flow rate of 5 L/ min, and the NC group was only exposed to distilled water. In the mixing chamber, sprayed PM_2.5_ was mixed with filtered clean air at a flow rate of 20 L/ min, and then the PM_2.5_ mixed with clean air was continuously sprayed into the whole-body exposure chamber at a flow rate of 25 L/min for 5 h (500 μg/m^3^ concentration in whole-body exposure).

### 4.5. Behavioral Tests

#### 4.5.1. Y-Maze Test

A Y-maze test was performed after PM_2.5_ exposure for 12 weeks. The Y-maze was made with black acrylic with three arms (33 l × 15 h × 10 w). The test was performed for 8 min, and mice movements were recorded using a Smart 3.0 video tracking system (Panlab, Barcelona, Spain).

#### 4.5.2. Morris Water Maze (MWM) Test

A MWM test was carried out for six days (visible platform (Day 1), hidden platform (Day two to five), and probe tests (Day six)) in a circular pool (150 cm in diameter). The pool was filled with water mixed with squid ink, and was separated into quadrants (N, S, E and W zones). The platform was positioned in the center of the W zone. Latency time to the hidden platform and mice movements were recorded using a video tracking system (maximum time: 1 min) during training. The hidden platform test was performed three times a day (Day 2–5). On day six (probe test), the learning and memory function was evaluated by measuring the time they stayed in the W zone. All experimental procedures were recorded using a Smart 3.0 video tracking system (Panlab, Barcelona, Spain) [69].

### 4.6. Mitochondrial Tests

#### 4.6.1. Isolation of Mitochondria

Brain tissue was homogenated with isolation buffer [215 mM mannitol, 75 mM sucrose, 0.1% BSA, 20 mM HEPES (Na^+^) and 1 mM ethylene glycol-bis(2-aminoethylether)-N,N,N′,N′-tetraacetic acid (EGTA)], using a bullet blender (Next Advance Inc., Averill Park, NY, USA). The homogenated brain was centrifuged (1300× *g*, 5 min, 4 °C). The supernatant was transferred to a new tube, and then centrifuged (13,000× *g*, 10 min, 4 °C), and the pellet was mixed with isolation buffer with 0.1% digitonin for 5 min, and added to the isolation buffer. Finally, the mixture was centrifuged (13,000× *g*, 15 min, 4 °C), and the pellet was re-mixed with the isolation buffer without 1 mM EGTA [70].

#### 4.6.2. Mitochondrial ROS Contents

The isolated brain mitochondria was incubated with DCF-DA (25 μM) staining solution for 20 min in a black 96-well plate. After 20 min of incubation, the fluorescent intensity was measured to excitation wave (485 nm and emission wave (535 nm) [70].

#### 4.6.3. Mitochondrial Membrane Potential (MMP)

The isolated brain mitochondria was incubated with the tetraethylbenzimidazolylcarbocyanine iodide (JC-1) dye in a black 96-well plate for 20 min. The fluorescence intensity was measured to excitation wave (535 nm) and emission wave (590 nm) [70].

#### 4.6.4. ATP Level

The ATP from mitochondria was extracted with 25 mM Tris-acetate buffer containing 1% trichloroacetic acid (pH 8.0) for 10 min in ice, and centrifuged (10,000× *g*, 15 min, 4 °C). The supernatant was used as the ATP extracts, and the level was measured using a commercial kit (Promega, Madison, WI, USA) with a luminescence meter (Promega).

### 4.7. MDA Content

The homogenized brains with PBS were centrifuged (6000× *g*, 10 min, 4 °C). The supernatant was added to 1% phosphoric acid and 0.67% 2-thiobarbituric acid. The mixtures were reacted using a water bath (95 °C, 1 h), and absorbance was measured at 532 nm using a microplate reader (Epoch 2, BioTek Instruments Inc., Winooski, VT, USA).

### 4.8. Western Blot Assay

The brains were homogenated with a RIPA buffer (ProtinEx^TM^ Animal cell/tissue, GeneAll Biotechnology, Seoul, Korea) containing 1% protease and phosphatase inhibitors, and centrifuged (13,000× *g*, 10 min, 4 °C). The extracted proteins were separated using sodium dodecyl sulfate polyacrylamide gel and transferred to a polyvinylidene difluoride membrane (Millipore, Billerica, MA, USA). The membranes were combined with the primary antibodies for at least 10 h at 4 °C after blocking using 5% skim milk. The protein-antibody complex was incubated with the secondary antibodies for 2 h and detected using a Chemi-doc iBright Imager (Thermo-Fisher Scientific, Waltham, MA, USA).

### 4.9. Measurement of Cytokine Content

The cytokines in the lungs, guts, blood serum and brain were analyzed using a mouse magnetic Luminex assay kit, and measured using the MAGPIX^Ⓡ^ instrument (Luminex Corporation, Austin, TX, USA) with xMAP technology and xPONENT 4.2 software.

### 4.10. Bioactive Chemical Analysis of P. tenera

#### 4.10.1. Molecular Weight Analysis

The average molecular weight was determined using gel permeation chromatography (HLC-8320, Tosoh Bioscience, Stuttgart, Germany), and was separated using TSKgel Guard PWxl+2xTSKgel GMPWxl+TSKgel G2500PWxl columns (7.8 × 300 mm, Tosoh Bioscience, Stuttgart, Germany) with 0.1 M NaNO_3_ for 60 min.

#### 4.10.2. Sulfate Contents

*P. tenera* was incubated in a water bath with 1 M HCl (105 °C for 5 h), and mixed with 3% TCA and a BaCl_2_-gelatin solution. The mixture was incubated for 15 min for the release of barium sulfate. The sulfate content was determined by measuring at 360 nm using a microplate reader (EPOCH2; BioTek, Winooski, VT, USA), and calculated by substituting the standard curve using potassium sulfate [71].

#### 4.10.3. Monosaccharide Composition

The monosaccharide constituents were analyzed using high-pH anion-exchange chromatography with a pulsed amperometric detection (HPAEC-PAD) system (Dionex, Sunnyvale, CA, USA). The monosaccharides were separated using a CarboPacTM PA1 column (25 cm × 4 cm, 0.4 × 25 cm, Dionex, Sunnyvale, CA, USA) with 18 mM and 200 mM NaOH for 15 min.

#### 4.10.4. Major Bioactive Compound Analysis

The major bioactive compounds were analyzed using the Waters Acquity UPLC-Q-TOF/MS system. The compounds were separated using an Acquity UPLC BEH C18 column (2.1 × 100 mm, 1.7 μm pore, Waters Corp, Milford, MA, USA) in positive ion mode. The following solvent gradients (mobile phase A: distilled water contained 0.1% formic acid and B: acetonitrile) were added as follows: 0 to 12.0 min, 0% to 80% B. The conditions for MS analyses were: drying gas (N_2_) temperature 120 °C, drying gas flow 30 L/h, nebulizer pressure 40 psi, fragmented voltage 175 V, capillary voltage 3 kV, and mass range from *m*/*z* 100 to 1500.

### 4.11. Gut Microbiome Analysis

The gut microbiota in feces was analyzed using next-generation sequencing (NGS) 16S rRNA sequencing analysis. Genomic DNA preparation and purification was experimented with a QIAamp DNA stool mini kit (Qiagen Canada, Mississauga, ON, Canada), and extracted DNA was used as template DNA for NGS analysis (MiSeq System, Illumina Inc., San Diego, CA, USA).

### 4.12. Measurement of SCFAs Contents

The feces were homogenated with NaOH (5 mM), and centrifugated (12,000× g, 10 min, 4 °C). The supernatant was added to propanol/pyridine (3:2, *v*/*v*) and propyl chloroformate, and the mixture was vortexed and sonicated. SCFAs were then extracted from the mixtures using *n*-hexane, and the *n*-hexane fraction was analyzed using a GC-MS-TQ 8030 triple quadrupole mass spectrometer (Shimadzu, Kyoto, Japan) and GC/MS-QP 2010 Plus (Shimadzu, Kyoto, Japan). The SCFAs were separated using a DB-5MS column (30 m × 0.25 mm, thickness 0.25 μm) with a flow rate of 1.0 mL/min, and the following GC analysis conditions: the initial temperature was 40 °C, which was maintained for 5 min, and was then allowed to reach 310 °C at a rate of 10 °C /min (split ratio 60:1, injection temp 200 °C, and column oven temp 60 °C) [19].

### 4.13. Statistical Analysis

All results were expressed as the mean ± standard deviation. The significant differences were analyzed by one-way analysis of variance with Duncan’s new multiple-range test (*p* < 0.05) of SAS ver. 9.4 (SAS Institute Inc., Cary, NC, USA).

## 5. Conclusions

The administration of *P. tenera* ameliorated PM_2.5_-induced learning and memory impairments, and alleviated PM_2.5_-induced oxidative stress and inflammation in the brain by regulating mitochondrial activation and the NF-κB/inflammasome mechanism. In addition, *P. tenera* attenuated Aβ production/tau phosphorylation through the regulation of JNK signaling. Furthermore, in the gut-brain axis, intake of *P. tenera* containing sulfated galactan, MAAs and chlorophyll derivatives as bioactive compounds attenuated damage to intestinal permeability and inflammation through changes in the gut microbiota, tight junction-related protein expression and SCFAs production in PM_2.5_-induced gut dysfunction. Therefore, *P. tenera* including sulfated galactan as prebiotics and MAAs as materials that pass the BBB is suggested as a potential material for cognitive function through the inhibition of inflammation and oxidative damage by PM_2.5_.

## Figures and Tables

**Table 1:** Total polysaccharide, average molecular weight and composition (*w/w*% of dried weight) of *Porphyra tenera*.

Total Polysaccharide (%)	Mw (kDa)	Sulfate (%)	Relative Area (%)					
			Fucose	Rhamnose	Galactose	Glucose	Xylose	Others
46.23 ± 0.39	220.49	43.25	6.52	7.83	44.24	-	23.18	18.23

**Table 2:** Identified compounds of *Porphyra tenera* by UPLC/MS Q-TOF system.

Retention Time (min)	ESI+ (*m/z*)	Collision Energy (eV)	Fragment Ion	Identified Compounds
0.83	347.1450	25	303, 244, 227, 200, 186	porphyra-334 isomer
1.50	347.1450	25	303, 244, 227, 200, 186	porphyra-334 isomer
4.03	285.1438	25	241, 197	palythene
4.54	329.1333	20	279, 253, 233, 205, 187, 150	palythenic acid
9.87	593.2760	40	533, 460, 447	pheophorbide a
